# Disease-Associated Plasmacytoid Dendritic Cells

**DOI:** 10.3389/fimmu.2017.01268

**Published:** 2017-10-16

**Authors:** Shuang Li, Jing Wu, Shan Zhu, Yong-Jun Liu, Jingtao Chen

**Affiliations:** ^1^Institute of Translational Medicine, The First Hospital, Jilin University, Changchun, China; ^2^Sanofi Research and Development, Cambridge, MA, United States

**Keywords:** plasmacytoid dendritic cells, dysregulation, malignancy, autoimmune disease, tumor microenvironment

## Abstract

Plasmacytoid dendritic cells (pDCs), also called natural interferon (IFN)-producing cells, represent a specialized cell type within the innate immune system. pDCs are specialized in sensing viral RNA and DNA by toll-like receptor-7 and -9 and have the ability to rapidly produce massive amounts of type 1 IFNs upon viral encounter. After producing type 1 IFNs, pDCs differentiate into professional antigen-presenting cells, which are capable of stimulating T cells of the adaptive immune system. Chronic activation of human pDCs by self-DNA or mitochondrial DNA contributes to the pathogenesis of systemic lupus erythematosis and IFN-related autoimmune diseases. Under steady-state conditions, pDCs play an important role in immune tolerance. In many types of human cancers, recruitment of pDCs to the tumor microenvironment contributes to the induction of immune tolerance. Here, we provide a systemic review of recent progress in studies on the role of pDCs in human diseases, including cancers and autoimmune/inflammatory diseases.

## Introduction

Plasmacytoid dendritic cells (pDCs) were first described in 1958 by pathologists Lennert and Remmele (1). Human pDCs are often identified and classified based on the coexpression of CD123 and CD303, whereas mouse pDCs express B220 and CD11c. pDCs recognize RNA and DNA viruses through toll-like receptor (TLR)-7 and -9, leading to activation of pDCs, and release high amounts of type I interferon (IFN-I) (2). Activated pDCs express high levels of major histocompatibility complex class II (MHC II), and costimulatory molecules (CD40, CD80, CD83, and CD86) enable pDCs to act as antigen-presenting cells to present antigens to CD4^+^ T cells. Moreover, pDCs secrete other proinflammatory cytokines and chemokines, such as interleukin-6 (IL-6), IL-12, CXC-chemokine ligand 8 (CXCL8), CXCL10, CC-chemokine ligand 3 (CCL3), and CCL4. Thus, the biology of pDCs is multifaceted (2, 3).

Plasmacytoid dendritic cells were originally derived from bone marrow hematopoietic stem cells (HSCs) (4). In the presence of Fms-like tyrosine kinase ligand (Flt3L) and Flt3L receptor signaling, HSCs could differentiate into pDCs and other DC subsets, such as conventional dendritic cells (5). In the presence of some special factors, such as IFN regulatory factor 8 (IRF8), E2-2, basic leucine zipper transcription factor ATF-like 3 (Batf3), and IFN-I, molecular signaling restricts the development of common lymphoid progenitor and common myeloid progenitor (CMP) lineages into pDCs (2, 6, 7). Recently, Reis and Sousa’s group demonstrated that besides CMP, human pDCs could also arise from multipotent lymphoid progenitors (MLPs). Compared with CMPs, MLPs show better potential for pDC production (8). The biology of pDCs is multifaceted, and analysis of the different origins of pDCs may help to explain the biology of pDCs and their functional heterogeneity in health and disease; however, further studies are needed to explore these aspects of pDC biology.

After leaving the bone marrow, blood pDCs directly migrate into primary lymphoid organs and reach T cell-rich areas of secondary lymphoid tissues *via* high endothelial venules (HEVs) in lymph nodes and mucosa-associated lymphoid tissues. Normally, pDCs are limited to primary and secondary lymphoid organs; however, under pathological conditions, functional chemotactic receptors expressed on circulating pDCs interact, and the corresponding ligands expressed by lymph nodes and non-lymphoid tissues facilitate pDCs trafficking to lymph nodes and diseased tissues through HEVs (9, 10).

## pDCs in Tumor Microenvironments

Malignant cells strongly interact with their microenvironment and modulate the cells in this niche to promote tumor growth and metastasis. The circulating pDCs recruited into the tumor microenvironment are characterized by decreased expression of costimulatory molecules and a reduced ability to produce IFN-I. Similarly, pDCs frequently display an inhibitory phenotype and promote a tolerogenic microenvironment through the activation of regulatory T cells (Tregs) (11). Malignant-derived immunosuppressive factors facilitate the infiltration of pDCs into disease tissue and interact with components derived from pDCs to inhibit the immune response. Tumor-associated pDCs then respond to malignant-derived immunosuppressive factors during the disease process through regulatory factors from TLR-7/9 signaling pathways and components produced by pDCs. Thus, pDCs promote tumor progression and attenuate immune regression (12, 13).

There are several mechanisms mediating the pathogenicity of disease-associated pDCs in different tumors. One of these mechanisms is inhibition of IFN-I, IL-6, tumor necrosis factor (TNF)-α, and IFN-inducible protein-10 (IP-10) release. Regulatory factors are expressed by pDCs *via* TLR-7/9 pathway, causing the signaling to proceed in the wrong direction and resulting in dysfunctional secretion of IFN-I e.g., IRF7 (14–16), indoleamine 2,3-dioxygenase (IDO) (17, 18), and immunoglobulin-like transcript 7 (ILT7) (19). In comparison, IFN-I secretion is also strongly disrupted by factors present in the disease microenvironment derived from necrotic cells or other immune cells, such as prostaglandin E2 (PGE2) (20), transforming growth factor beta (TGF-β) (21), IL-3 (22, 23), IL-10 (24), vasoactive intestinal peptide (VIP) (25), Wnt5a (26, 27), and high-mobility group box-1 protein (HMGB1) (28). In the second escape strategy, immunosuppression mediators decrease levels of costimulatory molecules and cause accelerated production of pDCs with immature characteristics, as demonstrated by VIP, Wnt5a, TNF-α, and HMGB1 (11). A third mechanism is *via* interactions between pDCs and other immune cells or malignant cells, wherein pDCs inhibit CD4 and CD8 T-cell proliferation and induce the differentiation of IL-10-producing T cells. Associated immaturity and coinhibitory molecules include IL-6, IL-8, CXCL12, HMGB1, IDO, ICOSL (29), granzyme B, OX40L, B-cell activating factor (BAFF), receptor activator of nuclear factor kappa B (RANK) (22), and granulocyte macrophage colony-stimulating factor (GM-CSF) (30).

In the following sections, we will discuss the functional significance of pDCs in various tumors and their role in mediating immunosuppression in the tumor microenvironment (Table 1). Thus, understanding the regulation of these mechanisms may contribute to the development of strategies to overcome tolerance in the tumor microenvironment.

**Table 1:** Changes in pDCs in different diseases.

Disease	Location	Number	Production of IFN-I	Upregulated molecules	Downregulated molecules	Upregulated chemokines	Reference
Melanoma	Tissue (LN/melanoma)	Increased	Decreased	IL-6, IDO, OX40L, ICOSL			(27, 31–33)
	Blood	Decreased		CD62L, CD86	CD80, CD83	CCR6, CXCR4, CXCR3, CCR7	(34)

Ovarian cancer	Ovary	Increased	Decreased	CD40, CD86	IFN-α, TNF-α, IL-6, MIP-1β, RANTES	CMKLR1, CXCR4	(35–37)

	Ascites	Increased		ICOSL	None	None	(37)
	Blood	Increased					
SLE	Skin	Increased	Increased			CCR9, Chemerin	(38)
	Blood	Decreased	Increased	IgE, CD123	HMGB1, CD80, CD86		(39–41)

Rheumatoid arthritis	Blood	Decreased	Decreased	IDO, IL-10	CD40, CD83, CD86, CD62L	CXCR3, CXCR4	(42, 43)
	Synovial fluids	Increased	Decreased		CD40, CD83, CD86, CD62L	CXCR3, CXCR4	(43)

Lung cancer	Lung	Increased		CD33, IL-1α, PD-L1	CD80, CD83	None	(44, 45)

Atherosclerosis	Plaques	Decreased	Increased	IDO, granzyme B	MHC II	None	(46–49)
					CD83		
	Blood	Decreased					(49)

*SLE, systemic lupus erythematosus; IDO, indoleamine 2,3-dioxygenase; ICOSL, inducible costimulator ligand; TGF-β, transforming growth factor beta; LN, lymph node*.

### Melanoma

Plasmacytoid dendritic cells have been shown to accumulate in the sentinel and metastatic lymph nodes in melanoma (31). Circulating pDCs from patients with melanoma have been found to express higher amounts of CCR6 and CXCR4, while their corresponding ligands CCL20 and CXCL12 are expressed on melanoma cells, suggesting that the CCR6/CCL20 and CXCR4/CXCL12 axes promote pDC migration from blood to melanoma foci (27, 34, 50, 51). Remarkably, CCL17, CCL22, and matrix metalloproteinase-2 found in the melanoma microenvironment have been shown to be associated with pDC accumulation (32). Some studies have also shown that pDCs migrating into the melanoma microenvironment are associated with early relapse and poor prognosis (26, 27, 30, 31).

**Figure 1:**
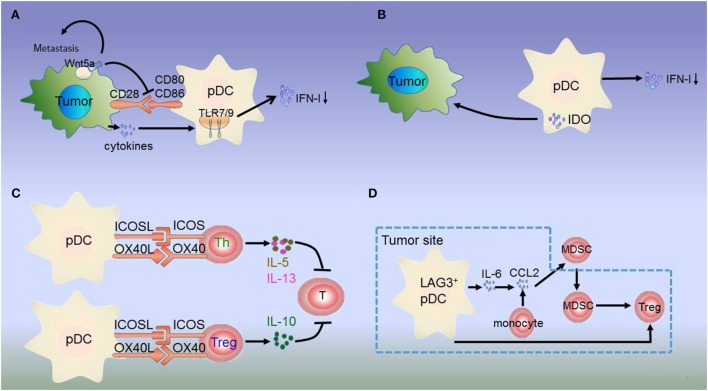
Dysregulation of plasmacytoid dendritic cells (pDCs) in melanoma. **(A)** Melanoma cells produce the immunosuppressive cytokines prostaglandin E2 (PGE2), interleukin-10 (IL-10), and transforming growth factor beta (TGF-β), which directly suppress type I interferon (IFN-I) production by inhibiting toll-like receptor (TLR) expression on pDCs. Additionally, melanoma-associated pDCs express Wnt5a, which blocks the upregulation of the activation markers CD80 and CD86 on human pDCs and inhibits toll-like receptor (TLR)-mediated pDC activation and production. Wnt5a can also promote melanoma metastasis. This inhibits antitumor function depending on IFN-I. **(B)** IDO expression in pDCs promotes immune evasion by the tumor. **(C)** Resident pDCs trigger IL-5/IL-13-secreting Th2 cells and IL-10-secreting Tregs through OX40L/OX40 and ICOSL/ICOS interactions. These cytokines inhibit cytotoxic T cell function and directly favor melanoma growth. **(D)** LAG3^+^ pDCs produce IL-6 without inducing IFN-I. pDC-derived IL-6 induces the production of CCL2—a key chemokine in the recruitment of myeloid-derived suppressor cells (MDSCs)—at the tumor site. LAG3^+^ pDCs and migratory MDSCs induce Tregs directly. Thus, through this alternative activation, LAG3^+^ pDCs promote immunosuppression.

Mediators of the tumor microenvironment act on tumor-infiltrating pDCs directly to suppress the production of IFN-I and mediate immunosuppression. Melanoma cells produce the immunosuppressive cytokines PGE2, IL-10, and TGF-β, which directly suppress IFN-I production by inhibiting TLR-7/9 and IRF7 expression on pDCs. Moreover, melanoma cells express Wnt5a, which inhibits TLR-mediated pDC activation and IFN-I production. Wnt5a potentiates melanoma metastasis *via* induction of the epithelial-to-mesenchymal transition in a protein kinase C-dependent manner (26, 27) (Figure 1A). IFN-I production may also be inhibited by ILT7, a ligand combined with BST2, which is expressed on melanoma cells. pDCs preferentially express ILT7, and the interaction between ILT7 and BST2 is involved in pDC and tumor crosstalk (52, 53).

Besides low IFN-α production, immunosuppressive mediators secreted by pDCs induce Tregs or suppress Th2 cell secretion to prevent an effective antitumoral response. pDCs in the tumor site express the immune-suppression molecules OX40L and ICOSL, which support melanoma progression (30). pDC infiltration is strongly associated with primary melanoma cell expression of activated signal transducer and activator of transcription 3, which is constitutively expressed in cancer and is thought to be a significant mediator of tumor-induced immunosuppression (31). Melanoma-associated pDCs have been shown to express high levels of IDO, suggesting that melanoma-derived signals may block pDC activation, thereby contributing to immune evasion (27, 31, 54, 55) (Figure 1B). Moreover, pDCs in the tumor microenvironment trigger IL-5/IL-13-secreting Th2 cells and IL-10-secreting Tregs through the expression of OX40L and ICOSL. These cytokines may inhibit cytotoxic T-cell functions and directly favor melanoma growth (32) (Figure 1C).

The interaction between tumor-infiltrating pDCs and other immune cells results in immunosuppression. MHC II molecules on melanoma cells bind to lymphocyte-activated gene 3 (LAG3) expressed on the surface of pDCs, resulting in their tolerogenic activation. Accordingly, LAG3^+^ pDCs display a slightly activated phenotype and produce IL-6 *in vivo*. IL-6 production by pDCs induces CCL2 production by monocytes. CCL2 is an essential chemokine that functions during the recruitment of myeloid-derived suppressor cells (MDSCs) to the tumor site. Hence, the recruitment of LAG3^+^ pDCs into the tumors and their activation in the absence of IFN-I production drive MDSC-mediated immune suppression (56, 57) (Figure 1D). As such, the counter-regulatory immune mechanisms in melanoma exhibit extensive signaling crosstalk. IDO is expressed by pDCs, whereas MDSCs, programmed death ligand 1 (PD-L1)^+^ T cells, and CTLA-4^+^ Tregs are strongly interconnected and associated with advanced disease and negative outcome. Thus, combination treatments targeting these markers can lead to a synergistic response (55, 58).

### Hematological Malignancies

Various types of leukemia, multiple myeloma (MM), and malignant lymphoma are collectively defined as hematological malignancies. Among these hematological malignancies, pDCs have been reported to be mostly associated with the pathophysiology of MM, chronic lymphocytic leukemia (CLL), and chronic myelomonocytic leukemia.

#### Multiple Myeloma

Plasmacytoid dendritic cells from patients with MM exhibit increased numbers in the bone marrow compared with those in normal donors; moreover, pDCs are more frequently localized in MM bone marrow than in MM peripheral blood, representing a functional impairment (22, 59). The interaction between pDCs (BDCA2) and MM cells (CD138) increases the production of cytokines and chemokines, which can not only prolong the survival of pDCs but also confer growth, survival, and drug resistance in MM cells (59). Finally, pDC-MM cells surface receptor-ligand interactions (BAFF/APRIL and RANK/RANKL) trigger MM cell growth/survival through the nuclear factor (NF)-κB pathway. Thus, cytokines, chemokines, and direct contact between pDC and MM cells may play critical roles in mediating pDC survival and MM cell growth (22). Ray et al. further demonstrated that treatment with a TLR-9 agonist restored the ability of MM patient-pDCs to stimulate T-cell proliferation and enhance the cytotoxicity of bortezomib (60).

Multiple myeloma cells produce low levels of IL-3. However, when cocultured with pDCs, IL-3 secretion is increased. *In vitro* and *in vivo* studies have revealed that IL-3 can prolong pDC survival (22). Ray et al. recently demonstrated that SL-401, a novel anti-IL-3R antibody, blocks pDC-induced MM cell growth by targeting pDCs (59). These studies therefore validated the targeting of pDC-MM interactions as a therapeutic strategy to overcome drug resistance in MM.

Microenvironmental interactions between pDCs and other immune cells could lead to a poor prognosis in MM and promote tumor cell growth and survival indirectly. Additionally, the increased numbers of pDCs in the bone marrow of MM patients enhances Th22 cell polarization through TNF-α and IL-6 secretion. Th22 cells contribute to the increased abundance of IL-22^+^/IL-13^+^ T cells, thereby leading to poor prognosis in MM based on the effects of pDCs in the tumor microenvironment (61).

#### Chronic Lymphocytic Leukemia

Lower numbers of pDCs cells are found in the peripheral blood and bone marrow of patients with progressive CLL and functional impairments (62, 63). IFN-I production is attenuated owing to decreased TLR-9 expression by pDCs, resulting in dampened effector immune cell activity (63). In addition, factors derived from the tumor microenvironment facilitate the dysfunction of pDCs in CLL. Vascular endothelial growth factor (VEGF) receptor neuropilin-1 (NRP1) is a critical link between angiogenesis and immune tolerance. VEGF overexpression has been established in CLL and has been shown to stimulate higher NRP1 expression. Accordingly, the expression of NRP1 has been found to be considerably higher in pDCs from patients with CLL compared with those in healthy volunteers. This increased NRP1 expression mediates tumor escape from immune surveillance (64, 65). However, few studies have assessed the relationships among pDCs, tumor cells, and other immune cells in CLL, and more in-depth investigations are needed to explore these mechanisms further.

### Breast Cancer and Ovarian Cancer

Breast cancer and ovarian cancer frequently occur in women. Most patients present with metastases, leading to increased numbers of dysfunctional pDCs at both primary and metastatic sites, such as the bone or enterocyte (28). pDC infiltration in primary localized breast cancer is correlated with poor survival, suggesting that these immune cells may contribute to tumor progression and tumor metastases (35, 66).

#### Breast Cancer

In breast cancer, pDCs exhibit a slightly activated phenotype and produce decreasing amounts of IFN-I after TLR activation *in vitro* compared with pDCs from healthy human peripheral blood. One study showed that the synergistic response of TGF-β and TNF-α is an important *in vivo* mechanism blocking IFN-I production by tumor-associated pDCs through the inhibition of IRF7 signaling and nuclear translocation in gynecological malignancy. This finding indicated that targeting tumor-associated pDCs to restore their IFN-α production may be a promising strategy, achieved by combining TLR-7/9-based immunotherapy with TGF-β and TNF-α antagonists, in breast cancer (67). Interestingly, partial tumor-associated pDCs cause selective suppression of IFN-I production and possess the unique capacity to sustain the expansion of FoxP3^+^ Treg cells, which may contribute to breast cancer progression (68).

Certain relevant mechanisms of pDCs not only depend on IFN-I but also function as immunosuppression mediators to induce tumor progression through the receptor/ligand axis. In breast carcinoma, tumor-associated pDC expression of ICOSL drives the expansion and suppressive function of ICOS^+^ Tregs, leading to preferential accumulation of this Treg subset in the close vicinity of pDCs in the tumor microenvironment and a secretion of the immunosuppressive mediator IL-10 by stimulation with tumor-associated antigen (TAA). These results showed that the ICOS/ICOS-L interaction is a central event in immunosuppression of tumor-associated T cells. Thus, the infiltration of pDCs in neoplastic lesions favors the establishment of a tumor microenvironment by the activation and expansion of ICOS^+^ Tregs, accelerating disease progression (13, 69). Another crucial factor inducing tumor progression in breast cancer is GM-CSF, *via* the GM-CSF/pDC axis. GM-CSF produced by primary breast tumor cells induces the activation of pDCs expressing the GM-CSF receptor. The GM-CSF/pDC axis is also significantly associated with more aggressive breast cancer subtypes (30).

Breast cancer differs from other malignancies in its specific dissemination pattern. In breast carcinoma, increasing infiltration of pDCs is related to high levels of IL-3, IL-6, IL-10, IL-15, IP-10, monocyte chemotactic protein-1, and RANTES (66, 70). Besides being immunosuppressive, these chemokines and cytokines are known to directly or indirectly induce tumor metastasis. These soluble factors induce the expression of RANKL, which is important for osteoclast-mediated bone resorption, thereby helping metastatic cells to grow (66, 71). Upon breast cancer dissemination, there is a steady increase in pDCs numbers within the bone, resulting in a sustained Th2 response along with elevated levels of Tregs and MDSCs. Subsequently, pDCs and CD4^+^ T cells produce osteolytic cytokines and cause severe bone damage (66, 72, 73).

#### Ovarian Cancer

In ovarian cancer, pDCs were reported to be attracted to primary ovarian cancer and ascites through stromal-derived factor-1 (SDF-1)/CXCL12 (36, 74, 75). In addition, SDF-1 attracts pDCs to the tumor environment, where they induced angiogenesis through production of TNF-α and IL-8 to promote ovarian tumor angiogenesis (74).

Gilliet et al. found that the numbers of Foxp3^+^ Tregs accumulating in the tumor microenvironment of ovarian cancers could be attributed to the expression of ICOS on Tregs. Moreover, the expansion and suppressive functions of Tregs are strictly dependent on ICOSL costimulation provided by tumor-associated pDCs. Accordingly, ICOS^+^ Tregs were found to localize near tumor pDCs, and the number of Tregs is directly correlated with the numbers of pDCs in the tumors. These findings suggest an important role for the interaction between ICOSL^+^ pDCs and ICOS^+^ Foxp3^+^ Tregs, leading to tumor progression in ovarian cancer (35). Another Treg subset in ovarian cancers induced by pDCs is CD8^+^ Tregs. CD8^+^ Tregs significantly suppress myeloid dendritic cell-mediated TAA-specific T cell effector functions through IL-10 (36). In general, breast cancer and ovarian cancer have similar mechanisms for both tumor promotion and metastases.

Interestingly, a clinical trial indicated that pDCs might have a subtle relationship with sex (76–79). The production of IFN-α in response to pDCs *via* TLR-7 activation is higher in the presence of estrogens, indicating that estrogens may be an attractive target for specific regulation of this pathway (80). A recent study suggested that estrogens regulate pDC IFN-I production through IRF5, which may act by enhancing IFN-α production in synergy with IRF7 (81). Besides the role of estrogens, X-linked genetic factors could also be involved in the sex-dependent differences in the TLR-7-mediated responses of pDCs. The *TLR-7* gene is located on the X chromosome. While no sex-based biases have been observed linking pDCs with neoplastic disease, these cells are significantly associated with more aggressive gynecological carcinomas (30, 37).

### Hepatocellular Carcinoma (HCC)

Hepatocellular carcinoma is the most common type of liver cancer; however, the role of pDCs in HCC is not clear. Recent studies have demonstrated that the numbers of pDCs are increased in tumor tissue and decreased in blood of patients with HCC (82, 83), suggesting that peripheral pDCs migrate to liver lesions in patients with HCC. In addition, pDCs exposed to tumor-derived factors would enhance IL-10 production by CD4^+^ Tregs through upregulation of ICOSL (83). This can help tumor cells escape the immune system. *In vitro*, HSC-derived pDC-based vaccines are highly potent inducers of tumor-reactive T-cell and NK cell responses (84). These finding may provide insights into appropriate immunotherapies for HCC using pDCs.

### Gastrointestinal Cancer (GC)

To date, few studies have explored the role of pDCs in GC. Yu et al. found a positive correlation between pDCs and ICOS^+^ Tregs in peripheral blood and peritumor tissue from patients with GC (12). Additionally, Yang et al. demonstrated that CD123^+^ pDCs in tumor tissue and tumor draining lymph nodes may contribute to Treg development and promote tumor tolerance in the colorectal cancer (CRC) tolerogenic milieu (85). Briefly, pDCs play a potential role in recruiting Tregs, and both participate in the immunosuppression microenvironment of GC and CRC.

### Lung Cancer

Studies of pDCs in lung cancer have mostly focused on non-small cell lung carcinoma (NSCLC). The proportion of pDCs is significantly increased in the peripheral blood and tumor tissues of patients with NSCLC (44, 45).

In NSCLC, pDCs show immunosuppressive phenotypes, as determined by higher levels of CD33 and PD-L1. Based on the characteristics of lung tumor-associated pDCs, pDCs are able to produce high levels of IL-1α in an AIM2-dependent manner, facilitating tumor cell proliferation in the lung (45). Moreover, a study of pDCs in NSCLC patients with different clinical stages demonstrated that elevated pDC numbers were observed in cases with higher disease stages (III/IV) compared with those in cases with lower stages (I/II) (44), suggesting a close relationship between tumor-associated pDCs and tumor progression (86). Interestingly, patients with NSCLC who smoke exhibited elevated pDC numbers compared with those of nonsmokers (44). Exploring the role of pDCs in lung cancer may lead to the development of novel therapeutic strategies.

## Autoimmune Disease

Aberrant pDC function has been shown to be involved in psoriasis, systemic lupus erythematosus (SLE), and rheumatoid arthritis (RA). Autoimmune disease arises from an abnormal immune response of the body against certain substances and normal tissues under physiological conditions. Notably, peripheral pDCs and conventional DCs are significantly reduced in patients with autoimmune diseases (87–91); however, an increased number of pDCs has been found in human tissue lesions (92, 93). These findings could be attributed to the role of pDCs in inflamed tissue; in autoimmune disease, these cells are recruited from the blood (94). pDCs are normally absent from the skin. However, they accumulate in inflammatory dermatoses from peripheral blood, where they organize local immune responses (94–96).

Several chemokines expressed on pDCs have been shown to participate in migration from the peripheral blood to tissue lesions. In psoriasis and SLE, chemerin is abundantly produced by HEVs in reactive lymph nodes, whereas skin-infiltrating pDCs strongly express ChemR23 in pathological conditions. pDCs may be recruited to disease foci through the chemerin/ChemR23 axis (97–99). Moreover, in RA, CXCR3, CXCR4, and CCR7 are expressed on both blood-derived pDCs and synovial tissue-derived pDCs. Their corresponding ligands, CXCL-10, CXCL-11, and CXCL-12, are present in RA and multiple sclerosis and stimulate the chemotaxis of blood-derived pDCs (43, 100, 101). Here, we discuss the roles of pDCs in SLE and other autoimmune diseases, include psoriasis, RA, and type I diabetes (T1Ds).

### Systemic Lupus Erythematosus

**Figure 2:**
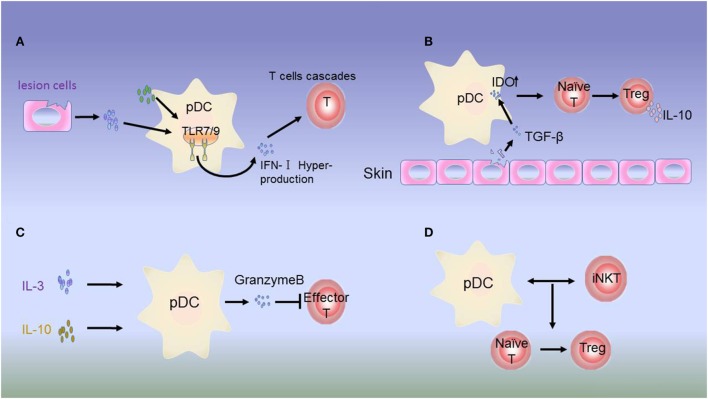
Dysregulation of plasmacytoid dendritic cells (pDCs) in autoimmune disease. **(A)** Hyperproduction of type 1 interferon (IFN-I) by pDCs. In psoriasis, systemic lupus erythematosus (SLE), and most autoimmune inflammations, constitutive expression of toll-like receptor (TLR)-7/9 leads to excessive activation of pDCs in the skin of patients by molecules from lesion cells or autologous pDCs. IFN-I produced by pDCs initiates abnormal production of T cells. **(B)** Immunosuppressive role of IDO. The inhibitory molecule IDO acts as a signaling protein in response to TGF-β, inducing the conversion of naive CD4^+^ and CD8^+^ T cells into Tregs. IDO can also dampen the antigen-presenting ability of pDCs. **(C)** In JIA, Tregs secrete low levels of IL-3 and IL-10. In response to these regulatory molecules, pDCs produce granzyme B to suppress the proliferation of effector T cells. **(D)** In type I diabetes (T1D), the interactions between pDCs and iNKT cells induce the T cells to differentiate into Foxp3^+^CD4^+^ Tregs.

In autoimmunity, pDCs may exhibit both immunogenic and tolerogenic functions according to the development of inflammatory autoimmune disorders (102). Accumulating evidence has suggested that pDCs can aggravate disease development in autoimmune disease, and this immunogenic function appears to be mediated partly by the overproduction of the inflammation-specific cytokine IFN-I (39, 103). IFN-I hyperproduction by pDCs is involved in the pathogenesis of SLE (104) (Figure 2A). Accordingly, the production of IFN-I depends on changes in the functions of mediators derived from autologous pDCs and lesion cells. For example, activation of IFN-I occurs *via* HMGB1 secreted by necrotic cells and inflammatory cells. HMGB1 then interacts with the receptor for generation of advanced glycation end products, which induces the activation of pDCs *via* the TLR-9-MyD88 pathway (105).

In addition, IFN-I overproduction may induce feedback regulation to target pDCs or other immune cells. In healthy individuals, pDCs promote the differentiation of immature B cells into IL-10-producing Bregs with stimulation by high concentrations of IFN-α, which subsequently suppresses IFN-α production by pDCs *via* IL-10 release. In SLE, despite aberrant Breg function, IFN-α overexpression may still result in regulatory feedback between pDCs and Bregs. Thus, altered pDC-Breg interactions contribute to the pathogenesis of SLE (106). IFN-I can also upregulate serum lupus autoantigens, such as Ro52 and laminin-1b, which could influence SLE progression (107, 108). Therefore, analysis of the functions of pDCs in SLE can help to develop new therapeutic strategies.

### Other Autoimmune Diseases

Interferon I production by pDCs also plays an immunogenic role in other autoimmune diseases. LL37, an antimicrobial peptide that is highly expressed in psoriatic lesions, binds self-DNA to form aggregated and condensed structures that are potent activators of pDCs *in vitro*. These complexes are delivered to pDCs to trigger TLR-9 expression and local IFN-I production (109). As a result, IFN-I released from pDCs in tissue lesions initiates the autoimmune T-cell cascade, facilitating autoimmunity (110).

In contrast to the immunogenic function of pDCs, increasing evidence has supported that unstimulated or alternatively stimulated pDCs can act as tolerogenic cells in autoimmune disease. Siglec-H is a surface molecule specifically expressed on mouse pDCs. Siglec-H-mediated antigen delivery was found to induce a hyporesponsive state in CD4^+^ T cells, leading to reduced expansion and inhibition of Th cell-dependent immunity (111). In addition, in the disease microenvironment, IL-3 and CD40L can activate pDC precursors, which are able to induce the differentiation of IL-10-producing CD8^+^ Tregs (112–114). In patients with juvenile idiopathic arthritis and cutaneous lupus erythematosus, pDCs can secrete large amounts of granzyme B in response to immunomodulatory cytokines, such as IL-3, IL-10, and IL-21. Moreover, pDC-derived granzyme B suppresses T-cell proliferation in a cell contact-dependent manner, similar to Tregs (115, 116) (Figure 2C). IDO can suppress the antigen-presenting ability of pDCs (117). In addition, studies have demonstrated that IDO can induce tolerogenic pDC function, although the underlying mechanism needs to be evaluated in animal models (42, 118) (Figure 2B).

Plasmacytoid dendritic cell function is tightly regulated in immune disorders by iNKT cells. pDCs are an essential partner of iNKT cells in T1D. Upon viral infection, iNKT cells induce TGF-β-producing pDCs in the pancreatic lymph nodes. These tolerogenic pDCs convert naive anti-islet T cells into Foxp3^+^CD4^+^ Tregs in pancreatic lymph nodes. Tregs are then recruited to pancreatic islets, where they produce TGF-β, which dampens the activity of viral- and islet-specific CD8^+^ T cells, thereby preventing T1D development in animal models (119–121) (Figure 2D).

Plasmacytoid dendritic cells exhibit different functional mechanisms during the development of inflammatory autoimmune disorders. Immunogenic pDC functions contribute to disease pathogenesis, e.g., SLE, through IFN-I production; however, tolerogenic pDCs may promote self-antigen-specific CD4^+^ T-cell tolerance and induce Treg differentiation, as observed in RA and T1D. Further studies of pDCs functional mechanisms in different diseases may facilitate the development of novel therapies.

## Therapeutic Prospects

Plasmacytoid dendritic cells induce immunosuppression and immune tolerance, thereby promoting disease progression. Potential solutions for disruption of tolerance include controlling IFN-I production by blocking IDO or TLR pathway activation or suppression of other inhibition molecules. IDO is highly expressed on pDCs when stimulated with IFN-I and TLR agonists in leukemia. Yamahira et al. investigated the effects of a novel IDO inhibitor, Toho-1, and found that it is efficient for potentiating antigen presentation of pDCs and may be applicable for pDC-based immunotherapy in tumors and severe viral infections (122).

Plasmacytoid dendritic cells are also thought to be involved in the pathogenesis of tumor and autoimmunity characterized by IFN-I *via* TLR-7/9 ligands in breast cancer and melanoma (123, 124). In melanoma treatment using the TLR-7 agonist imiquimod, infiltrating pDCs are capable of producing IFN-α and inducing complete regression or significant reduction of melanomas (11, 123, 125–127). Moreover, the TLR-9 agonist CpG activates the TLR signaling pathway and inhibits tumor growth in both breast cancer and melanoma mouse models (76, 124, 128). After activation of the TLR-7/9 pathway, pDCs promote the secretion of cytokines and initiate the activation of NK cells and CD8^+^ T cells. Synthetic TLR-7/9 agonists as adjuvants to cancer vaccines are currently being tested in human clinical trials and in combination with conventional chemotherapy and other protocols (Table 2).

**Table 2:** Clinical trials of pDCs in various diseases.

Target	Disease	Progress	Phase	Clinical trial no.	Combination
pDC vaccination	Prostatic neoplasms	Recruiting	II	NCT02692976	mDCs
	Melanoma	Recruiting	II	NCT02574377	mDCs
	Melanoma	Completed	I	NCT01690377	mDCs
TLR-7 agonist	Cancer, melanoma	Ongoing	II	NCT00960752	

A previous report demonstrated that targeting pDCs with nanoparticles *via* the C-type lectin DEC-205, DC immunoreceptor, blood DC Ag-2, or the FcR CD32 led to uptake, processing, and (cross-)presentation of encapsulated Ag to both CD4^+^ and CD8^+^ T cells. Thus, these receptors may be viable candidates to target pDCs with nanocarriers. pDCs induce potent antitumor responses because of their crosspresentation capacity (129, 130).

Plasmacytoid dendritic cells produce a systemic type I IFN response, which is critical to NK activation and subsequent inhibition of tumor metastasis. When compared with pDCs isolated from peripheral blood, *in vitro* differentiated pDCs exhibit an increased capacity to induce NK cell-mediated killing in acute lymphoblastic leukemia (131). Moreover, mDCs and pDCs have also been successfully utilized in combination in clinical vaccination trials against melanoma, wherein both mDCs and pDCs were found to enhance NK cell cytotoxicity to reach optimal activity (132, 133). Combination vaccination with distinct DC subsets may be required to simultaneously promote CD4^+^, CD8^+^ T-cell, and NK-cell responses (134).

### Clinical

Dendritic cell-based vaccines against cancer have also been developed during the past two decades. Clinical evidence showed that TAA-derived peptides loaded onto pDCs or CD1c^+^ DCs achieve promising efficacy in patients with melanoma (135, 136). Confronting immune checkpoint inhibitors targeting CTLA4, PD1, and PD-L1 may lead to clinical benefits in patients with various types of cancers, and next-generation DC vaccines are expected be developed by integration of DC-based vaccines with combinatorial immunotherapy regimens (137).

Besides potential therapeutic applications in cancer, pDCs have also been shown to have important properties in autoimmune diseases. For example, pDCs are major immune contributors in lupus *via* IFN-I overexpression. Early studies have shown that congenic lupus-predisposed mice lack pDCs because of IRF8 deficiency or SLC15A4 mutation. Moreover, the results indicated absence of autoantibodies, reduced lymphadenopathy and splenomegaly, and extended survival. IRF8 and SLC15A4 may therefore be important targets for therapeutic intervention in lupus (40). Notably, both IRF8 and SLC15A4 are upstream molecules of the TLR pathway and are important for IFN secretion. As abnormal pDC activation and deregulated IFN-I production appear to be contributing factors in autoimmune disease pathogenesis, future studies should be performed to determine whether IFN-I blockade or pDC depletion would be an effective method for the treatment of autoimmune diseases (138).

Recent findings of DCs could make us redefine our perception of DC populations. For example, See’s group defined a population of CD123^+^CD303^+^CX3CR1^+^CD33^+^ cells as DC precursors (pre-DCs), which share surface markers with classically defined pDCs and exhibit distinct functional properties. After removal of pre-DCs from the classically defined “pDC” population, the induction of T-cell proliferation and production of T-cell stimulatory ligands by pDCs is decreased (139). Additionally, Villani et al. demonstrated six DC and four monocyte cell clusters using single-cell RNA-sequencing. The authors validated the presence of Axl^+^Siglec6^+^ DCs (AS DCs), which share transcriptional modules with classically defined “pDCs,” but do not secrete IFNα and have stronger capacity to activate T cells (140). To date, pre-DCs and AS DCs have been shown to have similar functions; therefore, further studies are needed to verify their identities. The discovery of these two subtypes improves our understanding of classically defined pDCs, which should be reconsidered based on the antigen presenting and cytokine-secretion functions of pDCs; the taxonomy may also need to be revised accordingly. These concepts are expected to facilitate more precise analyses of DC subset-specific targeting in health and disease.

Plasmacytoid dendritic cells are thought to be involved in the pathogenesis of a variety of diseases. Given the capacity of pDCs to easily switch phenotypes and functions according to disease microenvironmental signals, this plasticity may be harmful when disorders occur in individuals. In this review, we summarized the possible mechanisms of deterioration induced by pDCs. Different immunotherapeutic approaches, as well as combinations with other local or systemic disease therapies, may be required to realize synergistic benefits. Immunotherapy induced by pDCs will cover many nonclassic diseases. However, further clinical trials are necessary to identify the effective dose and criteria for suitable patients.

## Author Contributions

SL and JW carried out the primary literature search, wrote and revised the manuscript, created the illustrations, and contributed equally to the review. SZ and Y-JL involved in the preparation and revision of manuscript. JC initiated the concept and supervised the manuscript writing and revision. All authors read and approved the final manuscript.

## Conflict of Interest Statement

Y-JL is employed by Sanofi Research and Development, USA. The role of this author was revising the manuscript. All authors declare no competing interests. The reviewer MH and handling editor declared their shared affiliation.
